# Supplementary material for: Low Mutational Burden of Extranodal Marginal Zone Lymphoma of Mucosa-Associated Lymphoid Tissue in Patients with Primary Sjogren’s Syndrome
Source: Cancers (Basel). 2022 Feb 17;14(4):1010. doi: 10.3390/cancers14041010 (PMC8870522; doi:10.3390/cancers14041010)
Supplement: Supplementary file 1 [file cancers-14-01010-s001.zip › cancers-1562681-supplementary.pdf]

cancers MDPI

Table S1. Patient characteristics.

Sample ID	Age (Years)	Gender	Tumor Sample	Ann Arbor Stage	RF	SS-A	SS-B	Cryoglobulinemia, Type
P1	49	Female	Fresh-frozen	1	119	>240	2	IgM, type 1, l
P2	40	Female	Fresh-frozen	1	52	>240	27	n.a.
P3	74	Female	Fresh-frozen	1	263	>240	1	n.a.
P4	63	Female	Fresh-frozen	1	48	>240	>320	n.a.
P5	59	Female	Fresh-frozen	1	16	77	13	IgG poly, type 3
P6	54	Female	Fresh-frozen	1	257	>240	117	IgM, type 2, k
P7	57	Female	Fresh-frozen	2	47	167	0	IgM, type 2, k
P8	84	Female	Fresh-frozen	2	>200	>240	>320	IgG poly, type 3
P9	69	Male	Fresh-frozen	1	30	>240	51	IgG poly, type 3
P10	74	Female	Fresh-frozen	4	9	>240	25	IgM, type 2, k + l
P11	60	Female	Fresh-frozen	2	114	>240	>320	IgM, type 2, k + l
P12	71	Female	Fresh-frozen	1	15	>240	23	IgM, type 1, k
P13	73	Female	Fresh-frozen	1	21	>240	41	n.a.
P14	62	Female	FFPE	2	26	>240	84	IgM, type 2, k
P15	64	Female	FFPE	2	>200	>240	>320	IgM, type 1, k
P16	55	Female	Fresh-frozen	1	2	205	0	IgM, type 2, l
P17	62	Female	FFPE	2	133	>240	1	IgM, type 2, k

RF indicates rheumatoid factor; SS-A, Sjogren's syndrome type A antigen and SS-B, Sjogren's syndrome type B antige.n

Table S2. Quality report of sequencing data generated by whole-exome sequencing for the 17 patients with primary Sjogren's syndrome related extranodal marginal zone lymphoma of mucosa-associated lymphoid tissue.

Sample ID	Reads Raw	Unique Reads Aligned	Mean Target Coverage	Fraction Bases 50x
P1T	59072934	53887968	121	97
P1B	60767448	55391891	125	97
P2T	71998200	65794494	140	97
P2B	65756454	59588099	136	95
P3T	63125628	57812755	130	97
P3B	58442296	53462456	121	97
P4T	55481062	47815146	97	95
P4B	58746888	53668388	118	96
P5T	62201224	56711290	128	97
P5B	67329370	61105560	138	97
P6T	64391210	58733639	133	97
P6B	63277434	57666554	130	97
P7T	63630990	58237896	131	97
P7B	62640026	57001533	130	97
P8T	57333826	53306257	119	97
P8B	73024826	66420293	150	97
P9T	59521570	54480511	120	96
P9B	57710138	53225428	118	97
P10T	63231656	57746559	131	97
P10B	65524444	59422786	133	98
P11T	63690470	58477017	128	97
P11B	57982854	53077083	117	96
P12T	43369524	40049442	88	93
P12B	70326286	65171493	144	98
P13T	68897064	63382909	140	97
P14T	39590978	36444558	84	90
P15T	53446102	48876677	108	95
P16T	74128410	68287802	154	98

P17T	77904698	71240981	163	97

B indicates blood and T, tumo.r

Table S3. Somatic variants in the 12 cases with a matched blood sample.

	Variant Information									Read Counts in WBC				Read Counts in Tumor				Variant
Sample ID	Gene	HG19 Position	Reference Allele	Variant Allele	Type	Effect	A.a. Change	dbSNP_ID	CADD Score	REF	ALT	Depth	VAF	REF	ALT	Depth	VAF	Major/minor
P1	*MAMDC4*	chr9:139748661	A	C	SNV	splice_acceptor			33.0	30	3	33	9.1	26	6	32	18.8	major
P1	*SLC9B1*	chr4:103832643	A	C	SNV	missense	p.Leu294Arg	rs200256569	23.9	96	0	96	0.0	104	10	114	8.8	minor
P1	*AK2*	chr1:33478834	A	G	SNV	missense	p.Ile223Thr	rs758305242	26.7	98	3	101	3.1	117	11	128	8.6	minor
P2	*FANCM*	chr14:45605577	C	G	SNV	missense	p.Leu115Val		23.4	156	0	156	0.0	121	32	153	20.9	major
P2	*FAM24A*	chr10:124671176	C	T	SNV	missense	p.Thr9Met	rs143764952	15.4	105	0	105	0.0	94	24	118	20.3	major
P2	*SKP2*	chr5:36153084	G	A	SNV	missense	p.Gly74Arg		23.6	178	0	178	0.0	128	32	160	20.0	major
P2	*TBL1XR1*	chr3:176744225	C	T	SNV	missense	p.Gly485Glu		25.7	64	0	64	0.0	108	26	134	19.4	major
P2	*CLDN17*	chr21:31538395	G	T	SNV	missense	p.Leu181Met		19.2	144	0	144	0.0	129	29	158	18.4	major
P2	*BCL7A*	chr12:122460086	A	G	SNV	missense	p.Lys30Arg	rs750629218	15.8	86	0	86	0.0	41	9	50	18.0	major
P2	*DARS*	chr2:136690414	T	A	SNV	splice_acceptor			34.0	12	3	15	20.0	52	10	62	16.1	major
P2	*DNAH5*	chr5:13845104	T	G	SNV	splice_acceptor			31.0	77	2	79	2.5	44	7	51	13.7	major
P2	*CFL1*	chr11:65623567	CTCCAG-GATG ATGTTCTTCTTG	C	DELETION	inframe_deletion	p.Asp81_ Leu87del			209	0	209	0.0	121	18	139	12.9	major
P3	*KRT1*	chr12:53073763	T	A	SNV	missense	p.Ser124Cys	rs564926977	15.6	39	3	42	7.1	30	8	38	21.1	major
P3	*LYST*	chr1:235896969	T	G	SNV	missense	p.Lys2879Gln		24.0	96	0	96	0.0	75	17	92	18.5	major
P3	*BOD1L1*	chr4:13629119	G	GCTGGC	INSERTION	frameshift	p.Pro32fs			17	1	18	5.6	20	4	24	16.7	minor
P3	*MAMDC4*	chr9:139748661	A	C	SNV	splice_acceptor			33.0	28	2	30	6.7	29	5	34	14.7	minor
P3	*COG5*	chr7:107204427	CAG	C	DELETION	frameshift	p.Gly2fs	rs1430566056		56	2	58	3.4	40	5	45	11.1	minor
P3	*GAS6*	chr13:114566824	CCAG	C	DELETION	inframe_deletion	p.Leu23del	rs563012373	21.2	23	0	23	0.0	34	4	38	10.5	minor
P3	*TTC28*	chr22:29075636	G	T	SNV	missense	p.Pro26Thr	rs748336223	16.3	36	3	39	7.7	44	4	48	8.3	minor
P4	*TMEM2*	chr9:74300311	T	A	SNV	splice_acceptor			33.0	1	3	4	75.0	12	11	23	47.8	major
P4	*TTC28*	chr22:29075636	G	T	SNV	missense	p.Pro26Thr	rs748336223	16.3	31	2	33	6.1	19	17	36	47.2	major
P4	*HES1*	chr3:193855763	C	G	SNV	missense	p.Ala195Gly		22.6	44	2	46	4.4	14	7	21	33.3	major
P4	*GAPVD1*	chr9:128064260	A	T	SNV	splice_acceptor		rs777703562	35.0	15	1	16	6.3	15	5	20	25.0	major
P4	*TTBK1*	chr6:43252929	T	G	SNV	missense	p.Ser1261Thr		22.7	57	2	59	3.4	29	6	35	17.1	minor
P4	*DRG1*	chr22:31796701	GGGT	G	DELETION	inframe_deletion	p.Gly51del	rs770636655	21.5	35	0	35	0.0	22	4	26	15.4	minor
P5	*CPD*	chr17:28706626	A	T	SNV	missense	p.Asn210Tyr		26.2	47	1	48	2.1	29	8	37	21.6	major
P5	*CPD*	chr17:28706629	A	T	SNV	missense	p.Ser211Cys		25.1	47	1	48	2.1	30	8	38	21.1	major
P6	*KIFC2*	chr8:145693317	A	G	SNV	start_lost	p.Met1?	rs775189259	16.4	95	0	95	0.0	53	9	62	14.5	major
P6	*DGKI*	chr7:137531515	TGGC	T	DELETION	inframe_deletion	p.Ala31del	rs527683728	17.8	9	0	9	0.0	36	4	40	10.0	major
P7	*SRGAP1*	chr12:64491019	A	T	SNV	splice_acceptor		rs79989081	35.0	23	3	26	11.5	22	5	27	18.5	major

P7	*C5orf51*	chr5:41909846	A	T	SNV	splice_acceptor		rs199681766	34.0	26	3	29	10.3	23	5	28	17.9	major
P8	*METTL9*	chr16:21623964	A	T	SNV	splice_acceptor		rs754980571	35.0	11	3	14	21.4	13	7	20	35.0	major
P8	*MAPKAPK2*	chr1:206858639	A	C	SNV	missense	p.Gln22Pro		16.6	47	0	47	0.0	15	8	23	34.8	major
P8	*SKOR2*	chr18:44774746	A	C	SNV	missense	p.Val270Gly		16.0	78	3	81	3.7	38	11	49	22.4	major
P8	*PABPC3*	chr13:25671195	A	G	SNV	missense	p.Arg287Gly	rs201411821	22.5	258	1	259	0.4	172	4	176	2.3	minor
P9	*CAMSAP3*	chr19:7660896	A	G	SNV	missense	p.Glu3Gly		23.1	17	1	18	5.6	13	7	20	35.0	major
P9	*ADRA1B*	chr5:159399266	T	**A**	SNV	missense	p.Phe444Ile		22.6	43	1	44	2.3	15	7	22	31.8	major
P9	*SPINK13*	chr5:147661666	GC	G	DELETION	frameshift	p.Arg38fs			55	0	55	0,00	52	17	69	24.6	major
P9	*TTC28*	chr22:29075636	G	T	SNV	missense	p.Pro26Thr	rs748336223	16.3	21	2	23	8,70	39	7	46	15.2	minor
P9	*KRT18*	chr12:53343105	C	T	SNV	missense	p.Arg50Cys	rs78479490	22.6	62	3	65	4,62	65	11	76	14.5	minor
P9	*MUC4*	chr3:195505774	G	T	SNV	missense	p.Pro4226Leu		22.4	20	3	23	13,04	66	11	77	14.3	minor
P9	*FAM50A*	chrX:153678033	G	A	SNV	missense	p.Ala244Thr	rs370055697	24.5	1	46	47	2.1	6	60	66	9.1	minor
P10	*ANGPT1*	chr8:108509517	C	T	SNV	missense	p.Met90Ile		23.4	133	0	133	0.0	77	47	124	37.9	major
P10	*OR11A1*	chr6:29395328	T	TA	INSERTION	frameshift	p.Ile31fs	rs1276415425	17.4	207	1	208	0,5	101	59	160	36.9	major
P10	*CNGA1*	chr4:47939597	A	T	SNV	missense	p.Met374Lys		25.7	122	0	122	0.0	56	32	88	36.4	major
P10	*MAP1LC3C*	chr1:242159648	C	A	SNV	missense	p.Leu87Phe		23.9	123	0	123	0.0	96	52	148	35.1	major
P10	*SIPA1L2*	chr1:232538214	C	T	SNV	missense	p.Arg1649His	rs753321693	16.0	70	0	70	0.0	61	32	93	34.4	major
P10	*ID3*	chr1:23885707	G	A	SNV	stop_gained	p.Gln71*		49.0	125	0	125	0.0	99	51	150	34.0	major
P10	*MROH6*	chr8:144650826	C	T	SNV	missense	p.Gly514Ser		22.9	164	0	164	0.0	139	70	209	33.5	major
P10	*HMCN1*	chr1:186026388	C	G	SNV	missense	p.Asn2389Lys		23.6	121	0	121	0.0	82	41	123	33.3	major
P10	*IGLL5*	chr22:23230441	T	G	SNV	splice_donor			32.0	89	0	89	0.0	59	38	116	32.8	major
P10	*CCR6*	chr6:167550776	CAG	C	DELETION	frameshift	p.Glu354fs			133	0	133	0.0	58	28	86	32.6	major
P10	*TMED3*	chr15:79606114	C	T	SNV	missense	p.His62Tyr	rs1487873850	25.4	82	0	82	0.0	55	26	81	32.1	major
P10	*S100A2*	chr1:153534050	C	A	SNV	missense	p.Glu53Asp		22.8	126	0	126	0.0	85	40	125	32.0	major
P10	*GNB2*	chr7:100273798	A	C	SNV	splice_acceptor			33.0	22	3	25	12,0	22	10	32	31.3	major
P10	*DTX1*	chr12:113496084	G	C	SNV	missense	p.Glu29Asp		25.0	188	0	188	0.0	124	56	180	31.1	major
P10	*PAX9*	chr14:37132291	C	T	SNV	missense	p.Ser65Leu		32.0	163	0	163	0.0	116	52	168	31.0	major
P10	*TBL1XR1*	chr3:176763973	G	C	SNV	missense	p.Thr290Arg		31.0	52	0	52	0.0	25	11	36	30.6	major
P10	*SEC61A2*	chr10:12206392	A	T	SNV	missense	p.Tyr457Phe		20.7	166	0	166	0.0	99	43	142	30.3	major
P10	*CCT8L2*	chr22:17073298	C	T	SNV	missense	p.Arg48Gln	rs755816031	20.6	135	0	135	0.0	97	41	138	29.7	major
P10	*ID3*	chr1:23885800	AG	A	DELETION	frameshift	p.Ser39fs			165	0	165	0.0	125	51	176	29.0	major
P10	*RANBP2*	chr2:109381062	G	A	SNV	stop_gained	p.Trp1356*		38.0	150	0	150	0.0	98	40	138	29.0	major
P10	*PAX5*	chr9:37034003	G	A	SNV	missense	p.Thr9Ile		22.9	99	0	99	0.0	72	29	101	28.7	major
P10	*KLHL6*	chr3:183273269	A	G	SNV	missense	p.Leu58Pro	rs749398006	28.9	169	0	169	0.0	123	49	172	28.5	major
P10	*HNRNPR*	chr1:23637306	G	A	SNV	missense	p.Pro518Ser		18.8	124	0	124	0.0	78	31	109	28.4	major
P10	*CNTN3*	chr3:74411073	C	G	SNV	missense	p.Lys444Asn		22.5	134	0	134	0.0	104	41	145	28.3	major

P10	*LZTS2*	chr10:102766497	G	C	SNV	missense	p.Gly528Arg		22.7	172	0	172	0.0	127	50	177	28.2	major
P10	*ZBTB22*	chr6:33284023	G	C	SNV	missense	p.Ala224Gly		16.7	235	0	235	0.0	197	76	273	27.8	major
P10	*PLXNA4*	chr7:132193251	C	T	SNV	missense	p.Val68Ile	rs777642451	21.4	148	0	148	0.0	125	48	173	27.7	major
P10	*SCN8A*	chr12:52200324	T	A	SNV	missense	p.Met1685Lys		26.5	199	2	201	1,0	112	42	154	27.3	major
P10	*EARS2*	chr16:23540941	G	A	SNV	missense	p.Arg412Cys	rs549058278	24.3	63	0	63	0.0	59	22	81	27.2	major
P10	*PLXNC1*	chr12:94543091	GGGCGGCC	G	DELETION	frameshift	p.Ala116fs			104	0	104	0.0	83	31	114	27.2	major
P10	*DOCK10*	chr2:225672711	C	T	SNV	missense	p.Asp1168Asn		24.5	136	0	136	0.0	95	35	130	26.9	major
P10	*ID3*	chr1:23885625	G	A	SNV	missense	p.Pro98Leu		22.3	95	0	95	0.0	57	21	78	26.9	major
P10	*ERGIC3*	chr20:34144868	C	T	SNV	missense	p.Thr350Met		28.6	102	0	102	0.0	90	33	123	26.8	major
P10	*TMEM121*	chr14:105995464	AG	A	DELETION	frameshift	p.Ala99fs			131	0	131	0.0	90	33	123	26.8	major
P10	*ID3*	chr1:23885728	GC	G	DELETION	frameshift	p.Gln63fs			136	0	136	0.0	126	46	172	26.7	major
P10	*PPP2R5B*	chr11:64694340	G	A	SNV	missense	p.Gly119Asp		24.4	103	0	103	0.0	121	44	165	26.7	major
P10	*YTHDF3*	chr8:64099186	A	T	SNV	missense	p.Lys205Ile		27.9	159	0	159	0.0	85	31	116	26.7	major
P10	*KRTAP10-6*	chr21:46011728	C	A	SNV	missense	p.Ser213Ile		15.4	266	0	266	0.0	207	75	282	26.6	major
P10	*KCNJ3*	chr2:155555522	A	G	SNV	missense	p.Lys79Glu		24.6	173	0	173	0.0	107	38	145	26.2	major
P10	*TRIM55*	chr8:67061920	T	G	SNV	missense	p.Phe215Cys		29.1	124	0	124	0.0	76	27	103	26.2	major
P10	*SESN3*	chr11:94963972	G	A	SNV	missense	p.Thr18Ile		21.7	64	0	64	0.0	51	18	69	26.1	major
P10	*TMEM132D*	chr12:130184551	C	T	SNV	missense	p.Asp258Asn	rs1253234057	19.1	195	0	195	0.0	142	50	192	26.0	major
P10	*DGKB*	chr7:14724958	C	T	SNV	stop_gained	p.Trp247*		41.0	104	0	104	0.0	83	29	112	25.9	major
P10	*ESRP2*	chr16:68264750	TAG-TAGGCTGTGTAG-TTCAGGTAG	T	DELETION	frameshift	p.Leu633fs			123	0	123	0.0	81	28	109	25.7	major
P10	*IQUB*	chr7:123109369	T	A	SNV	stop_gained	p.Arg494*		39.0	132	0	132	0.0	92	31	123	25.2	major
P10	*SPRR3*	chr1:152975939	A	C	SNV	missense	p.Lys148Thr		15.5	166	0	166	0.0	113	38	151	25.2	major
P10	*MAGEB4*	chrX:30260899	G	A	SNV	missense	p.Arg216His	rs770408937	17.9	208	0	208	0.0	165	55	220	25.0	major
P10	*MMP3*	chr11:102713562	AT	A	DELETION	frameshift	p.Ile64fs		25.4	126	0	126	0.0	78	26	104	25.0	major
P10	*EMC4*	chr15:34517819	G	A	SNV	missense	p.Val57Met		22.9	103	0	103	0.0	76	25	101	24.8	major
P10	*UGT1A5*	chr2:234622076	G	A	SNV	missense	p.Asp147Asn		23.1	234	0	234	0.0	134	42	176	23.9	major
P10	*ZFPM2*	chr8:106814505	C	G	SNV	missense	p.Thr732Ser		22.8	138	0	138	0.0	102	32	134	23.9	major
P10	*IKZF2*	chr2:213886790	C	A	SNV	missense	p.Glu219Asp		25.1	137	0	137	0.0	115	36	151	23.8	major
P10	*TMEM121*	chr14:105995466	GGCGGCGCGGCGCGGC	GCCGCC	COMPLEX DELETION	frameshift	p.Ala99fs			135	0	135	0.0	93	29	122	23.8	major
P10	*BEX1*	chrX:102317883	G	T	SNV	missense	p.Ala107Glu		19.3	222	0	222	0.0	161	50	211	23.7	major
P10	*ARID2*	chr12:46123620	A	G	SNV	start_lost	p.Met1?		23.8	87	0	87	0.0	65	20	85	23.5	major
P10	*MPDZ*	chr9:13168465	C	A	SNV	missense	p.Asp1052Tyr		29.9	138	0	138	0.0	108	33	141	23.4	major
P10	*COL22A1*	chr8:139838993	A	G	SNV	missense	p.Tyr293His		26.3	100	0	100	0.0	86	26	112	23.2	major
P10	*MORF4L1*	chr15:79178554	T	C	SNV	missense	p.Leu115Pro		31.0	138	0	138	0.0	87	26	113	23.0	major

P10	*CCSAP*	chr1:229462505	C	T	SNV	missense	p.Ala206Thr		24.7	117	0	117	0.0	64	19	83	22.9	major
P10	*KIAA1109*	chr4:123107241	C	T	SNV	missense	p.His137Tyr		26.0	138	1	139	0,7	72	21	93	22.6	major
P10	*COL14A1*	chr8:121354624	C	A	SNV	missense	p.Asp1609Glu		23.6	131	0	131	0.0	86	24	110	21.8	major
P10	*RPS6KA2*	chr6:166862277	C	T	SNV	missense	p.Val448Met		22.4	124	0	124	0.0	98	27	125	21.6	major
P10	*SBNO2*	chr19:1109514	G	GA	INSERTION	frameshift	p.Ser1070fs			143	0	143	0.0	110	30	140	21.4	major
P10	*NAV3*	chr12:78574822	T	C	SNV	missense	p.Ser1897Pro		24.8	104	0	104	0.0	70	19	89	21.3	major
P10	*NFAT5*	chr16:69727535	GCAGCA GCAGCAA	G	DELETION	inframe_deletion	p.Gln1279_Gln1282del	rs747731827	18.2	98	0	98	0.0	112	30	142	21.1	major
P10	*DUSP5*	chr10:112258258	G	C	SNV	missense	p.Gly127Arg		34.0	113	1	114	0,9	87	23	110	20.9	major
P10	*LRRD1*	chr7:91793458	T	G	SNV	missense	p.Glu353Asp		24.1	86	0	86	0.0	70	17	87	19.5	major
P10	*BNIP2*	chr15:59981456	CC	TT	MNP	missense	p.GlyVal61GlyIle		16.2	183	0	183	0.0	106	25	131	19.1	minor
P10	*IGLL5*	chr22:23230321	G	A	SNV	missense	p.Gly30Ser	rs779042230	15.7	176	0	176	0.0	140	33	173	19.1	minor
P10	*KCTD8*	chr4:44177010	G	T	SNV	missense	p.Arg407Ser		22.5	171	0	171	0.0	128	30	158	19.0	minor
P10	*CENPI*	chrX:100387389	C	T	SNV	missense	p.Pro472Ser		20.1	145	0	145	0.0	95	22	117	18.8	minor
P10	*EIF4G1*	chr3:184033621	G	C	SNV	missense	p.Ala13Pro		23.0	60	3	63	4,8	49	11	60	18.3	minor
P10	*EIF4G1*	chr3:184033625	CAT	C	DELETION	frameshift	p.Pro14fs		32.0	56	3	59	5,1	42	9	51	17.6	minor
P10	*IGLL5*	chr22:23230441	T	A	SNV	splice_donor			32.0	89	0	89	0.0	59	20	116	17.2	minor
P10	*COL11A1*	chr1:103471837	G	A	SNV	missense	p.Thr585Met	rs202011565	18.9	82	0	82	0.0	89	18	107	16.8	minor
P10	*VCAN*	chr5:82849282	G	A	SNV	missense	p.Arg3198His	rs533779960	32.0	160	0	160	0.0	136	24	160	15.0	minor
P10	*HBP1*	chr7:106826262	A	AG	INSERTION	frameshift	p.Ile139fs			126	0	126	0.0	87	15	102	14.7	minor
P10	*DRC1*	chr2:26667615	T	C	SNV	missense	p.Trp399Arg	rs939820	16.2	1	98	99	1,0	5	141	146	3.4	minor
P11	*HS6ST3*	chr13:96743355	A	C	SNV	missense	p.Glu80Ala		16.4	16	3	19	15,8	10	13	23	56.5	major
P11	*IL10RA*	chr11:117869837	T	TC	INSERTION	frameshift	p.Gln407fs			147	0	147	0.0	113	50	163	30.7	major
P11	*RASA2*	chr3:141248642	C	T	SNV	stop_gained	p.Gln150*		45.0	97	0	97	0.0	57	23	80	28.8	major
P11	*NFATC4*	chr14:24837623	T	**G**	SNV	missense	p.Leu89Arg		24.4	20	1	21	4,8	25	10	35	28.6	minor
P11	*ID3*	chr1:23885715	T	C	SNV	missense	p.Glu68Gly		33.0	146	0	146	0.0	84	29	113	25.7	major
P11	*ID3*	chr1:23885464	C	G	SNV	missense	p.Ser116Thr		16.4	87	0	87	0.0	73	23	96	24.0	minor
P11	*NOTCH2*	chr1:120458047	G	A	SNV	missense	p.Ala2433Val		20.1	105	0	105	0.0	86	27	113	23.9	minor
P11	*COG3*	chr13:46039217	C	T	SNV	missense	p.Arg16Trp	rs564733357	25.0	71	0	71	0.0	55	17	72	23.6	minor
P11	*JAK1*	chr1:65309896	A	G	SNV	missense	p.Cys752Arg		15.7	64	0	64	0.0	88	27	115	23.5	minor
P11	*KCNK13*	chr14:90650784	T	A	SNV	missense	p.Trp222Arg		27.3	97	0	97	0.0	108	33	141	23.4	minor
P11	*IRX2*	chr5:2749046	T	G	SNV	missense	p.Lys259Thr		21.0	99	0	99	0.0	79	24	103	23.3	minor
P11	*GPR110*	chr6:46995492	A	AT	INSERTION	frameshift	p.Asn25fs		NA	71	0	71	0.0	52	15	67	22.4	minor
P11	*HNF4G*	chr8:76470798	G	T	SNV	missense	p.Ser250Ile		28.8	113	0	113	0.0	95	26	121	21.5	minor
P11	*ID3*	chr1:23885700	A	G	SNV	missense	p.Val73Ala		31.0	137	0	137	0.0	89	24	113	21.2	minor
P11	*TTC28*	chr22:29075636	G	T	SNV	missense	p.Pro26Thr	rs748336223	16.3	20	3	23	13,0	20	5	25	20.0	minor
P11	*DOCK5*	chr8:25149561	C	T	SNV	missense	p.Arg115Cys	rs760823537	26.0	104	0	104	0.0	74	18	92	19.6	minor
P11	*NFATC4*	chr14:24837623	T	A	SNV	missense	p.Leu89Gln		23.7	20	1	21	4,8	25	6	31	19.4	minor

P11	*HNF4A*	chr20:43030109	G	A	SNV	missense	p.Val33Met	rs1170574009	23.6	116	0	116	0.0	90	21	111	18.9	minor
P11	*ID3*	chr1:23885685	A	G	SNV	missense	p.Leu78Pro		32.0	127	0	127	0.0	92	21	113	18.6	minor
P11	*ZNF638*	chr2:71577318	A	G	SNV	missense	p.Met412Val	rs769864746	22.9	94	0	94	0.0	113	25	138	18.1	minor
P11	*IGLL5*	chr22:23230402	G	T	SNV	stop_gained	p.Gly57*		34.0	67	1	68	1,5	56	12	68	17.6	minor
P11	*UGT2A1*	chr4:70512759	C	T	SNV	missense	p.Asp202Asn	rs192398239	24.8	122	0	122	0.0	108	21	129	16.3	minor
P11	*POLR3A*	chr10:79764620	C	T	SNV	missense	p.Gly701Ser	rs142520086	26.4	122	0	122	0.0	92	17	109	15.6	minor
P11	*SLIT3*	chr5:168093559	C	T	SNV	missense	p.Arg1498His	rs140403495	28.9	117	0	117	0.0	94	17	111	15.3	minor
P11	*DLGAP3*	chr1:35331890	CCTT	C	DELETION	inframe_deletion	p.Lys911del	rs566251521	23.2	67	0	67	0.0	45	8	53	15.1	minor
P11	*FRMPD3*	chrX:106808247	G	A	SNV	missense	p.Arg449Gln	rs759785446	20.0	161	0	161	0.0	198	34	232	14.7	minor
P11	*LY6E*	chr8:144102363	ATCT	A	DELETION	inframe_deletion	p.Leu99del	rs752304887	16.5	133	0	133	0.0	113	19	132	14.4	minor
P11	*ATP10A*	chr15:25959016	G	A	SNV	missense	p.Arg717Trp	rs755196001	23.6	115	0	115	0.0	96	16	112	14.3	minor
P11	*WDR24*	chr16:739426	G	A	SNV	missense	p.Ser134Leu	rs1345675948	26.5	120	0	120	0.0	115	19	134	14.2	minor
P11	*KIAA1244*	chr6:138656236	G	A	SNV	missense	p.Glu2085Lys	rs1374975952	31.0	119	0	119	0.0	104	16	120	13.3	minor
P11	*ZEB2*	chr2:145157283	TGTGATAAC	T	DELETION	frameshift	p.Gly488fs			97	0	97	0.0	100	15	115	13.0	minor
P11	*SLC9B1*	chr4:103826769	G	A	SNV	stop_gained	p.Arg412*	rs200075071	36.0	97	2	99	2,0	132	16	148	10.8	minor
P11	*SLC9B1*	chr4:103826757	T	C	SNV	missense	p.Thr416Ala	rs879081874	15.0	97	2	99	2,0	134	15	149	10.1	minor
P12	*FZD2*	chr17:42635584	CG	C	DELETION	frameshift	p.Gly178fs	rs1221799995	24.6	31	0	31	0.0	17	4	21	19.0	major
P12	*CYP2A13*	chr19:41594994	A	G	SNV	missense	p.Tyr114Cys	rs763200548	21.9	82	2	84	2.4	33	6	39	15.4	major
P12	*AK2*	chr1:33479001	G	GATGTCATCTTTC	INSERTION	inframe_insertion	p.Met163_Asp166dup	rs1398317449	20.3	74	3	77	3.9	44	7	51	13.7	major
P12	*CYBA*	chr16:88709864	T	A	SNV	missense	p.Glu162Val		27.0	64	2	66	3.0	43	6	49	12.2	major
P12	*GCGR*	chr17:79766931	TTGC	T	DELETION	inframe_deletion	p.Leu17del	rs924505906	15.4	132	0	132	0.0	46	6	52	11.5	major
P12	*FZD3*	chr8:28385632	C	T	SNV	missense	p.Thr452Met	rs756134564	26.8	153	0	153	0.0	80	10	90	11.1	major
P12	*CYBA*	chr16:88709864	T	G	SNV	missense	p.Glu162Ala		24.4	64	2	66	3.0	43	5	48	10.4	major
P12	*CYP2A13*	chr19:41594916	A	G	SNV	missense	p.Lys88Arg	rs138627841	19.9	151	3	154	1.9	69	8	77	10.4	major
P12	*MST1*	chr3:49723321	AGCGCTG	A	DELETION	inframe_deletion	p.Gln406_Arg407del	rs2985131	21.6	108	2	110	1.8	74	8	82	9.8	major
P12	*CYP2A13*	chr19:41594891	G	A	SNV	missense	p.Val80Met	rs202058359	22.6	152	3	155	1.9	75	8	83	9.6	major

A.a. change indicates amino acid change; ALT, alternate allele; CADD score, Combined Annotation Dependent Depletion score; dbSNP ID, reference cluster ID; HG19, human genome version 19; MNP, multi-nucleotide polymorphism; REF, reference allele; SNV, single-nucleotide variant; VAF, variant allele frequency and WBC, white blood cell

Table S4. Somatic variants in the 5 cases without a matched blood sample.

		Variant Information								Read Count in Tumor				Variant
Sample ID	GENE_Symbol	HG19 Position	Reference Allele	Variant Allele	Type	Effect	a.a. Change	dbSNP_ID	CADD score	REF	ALT	Depth	VAF	Major/Minor
P13	*ELL*	chr19:18557592	G	C	SNV	missense	p.Pro500Ala	rs151228060	21.8	91	68	159	42.8	major
P13	*TRIM33*	chr1:115053650	GC	G	DELETION	frameshift	p.Gly16fs	rs1249422960		17	4	21	19.0	minor
P13	*ASPSCR1*	chr17:79954544	T	C	SNV	missense	p.Leu252Pro		24.9	40	4	44	9.1	minor
P14	*RNF213*	chr17:78325483	A	G	SNV	splice_acceptor		rs114046339	33.0	16	16	32	50.0	major
P14	*SETD2*	chr3:47103785	G	A	SNV	missense	p.Pro2054Leu	rs139016283	23.3	25	25	50	50.0	major
P14	*POLQ*	chr3:121209142	A	G	SNV	missense	p.Ile879Thr	rs145048812	26.3	40	39	79	49.4	major
P14	*NACA*	chr12:57114061	G	C	SNV	missense	p.Ser418Cys	rs150062953	22.4	36	37	73	49.3	major

p14	*ARID1A*	chr1:27107048	A	G	SNV	missense	p.Asn2220Ser	rs139576809	16.3	36	38	74	48.7	major
P14	*PIK3R1*	chr5:67576370	G	A	SNV	missense	p.Glu217Lys	rs540361957	24.3	14	15	29	48.3	major
P14	*CBLC*	chr19:45284517	T	C	SNV	missense	p.Val185Ala	rs200445340	23.9	40	37	77	48.1	major
P14	*CIC*	chr19:42794472	C	T	SNV	missense	p.Pro1427Ser	rs1270663092	20.4	34	38	72	47.2	major
P14	*MUC16*	chr19:8998761	G	A	SNV	missense	p.Pro13608Ser	rs766595157	15.8	34	38	72	47.2	major
P14	*FGFR2*	chr10:123353298	C	T	SNV	missense	p.Val12Met	rs143978938	15.1	42	37	79	46.8	major
P14	*WRN*	chr8:30989992	T	G	SNV	missense	p.Ile979Met	rs147802438	19.1	37	31	68	45.6	major
P14	*RAP1GDS1*	chr4:99363185	G	A	SNV	missense	p.Val582Ile	rs200477763	20.8	44	36	80	45.0	major
P14	*ABI1*	chr10:27052872	G	A	SNV	missense	p.Pro281Ser	rs149749581	22.5	29	37	66	43.9	major
P14	*APC*	chr5:112173884	C	T	SNV	missense	p.Pro865Ser	rs192620988	17.5	49	40	89	44.9	major
P14	*PCM1*	chr8:17872228	G	C	SNV	missense	p.Arg1907Pro	rs565494296	17.9	42	31	73	42.5	major
P14	*CBFA2T3*	chr16:88958819	G	A	SNV	missense	p.Pro152Ser	rs1406987240	23.8	68	50	118	42.4	major
P14	*TNC*	chr9:117783543	C	T	SNV	missense	p.Val2167Ile	rs149752009	23.6	19	13	32	40.6	major
P14	*MUTYH*	chr1:45797106	G	A	SNV	missense	p.Arg437Trp	rs587778540	22.1	35	23	58	39.7	major
P14	*CAMSAP3*	chr19:7660910	G	C	SNV	missense	p.Gly8Arg		22.4	13	8	21	38.1	major
P14	*NCOR1*	chr17:16089995	C	T	SNV	missense	p.Ala39Thr	rs150859090	21.3	27	16	43	37.2	major
P14	*HS6ST3*	chr13:96743355	A	C	SNV	missense	p.Glu80Ala		16.4	24	6	30	20.0	minor
P14	*HS6ST3*	chr13:96743363	C	G	SNV	missense	p.Arg83Gly		22.1	25	5	30	16.7	minor
P14	*MAMDC4*	chr9:139748661	A	C	SNV	splice_acceptor			33.0	21	4	25	16.0	minor
P14	*BCR*	chr22:23654017	G	A	SNV	missense	p.Asp1106Asn	rs879255379	26.3	49	8	57	14.0	minor
P15	*FAT1*	chr4:187629648	G	A	SNV	missense	p.Ala445Val	rs528553183	23.3	50	27	77	35.1	major
P15	*CLTCL1*	chr22:19223274	A	G	SNV	missense	p.Val305Ala	rs556526468	23.2	47	23	70	32.9	major
P15	*HES1*	chr3:193855774	G	C	SNV	missense	p.Gly199Arg		23.0	73	23	96	24.0	minor
P15	*HS6ST3*	chr13:96743363	C	G	SNV	missense	p.Arg83Gly		22.1	22	5	27	18.5	minor
P15	*TNFAIP3*	chr6:138198371	ACTCAT	A	DELETION	frameshift	p.Leu324fs			56	11	67	16.4	minor
P15	*HNF1A*	chr12:121432119	C	G	SNV	missense	p.Pro289Arg	rs267603343	22.3	56	4	60	6.7	minor
P15	*ESR1*	chr6:152265443	A	G	SNV	missense	p.Lys299Arg	rs77797873	22.8	77	4	81	4.9	minor
P16	*TMEM2*	chr9:74300311	T	A	SNV	splice_acceptor			33.0	7	14	21	33.3	major
P16	*BAX*	chr19:49458192	G	T	SNV	missense	p.Gly3Trp	rs553879570	29.2	60	28	88	31.8	major
P16	*HOXA13*	chr7:27239672	G	A	SNV	missense	p.Pro9Ser	rs570503434	24.0	53	24	77	31.2	major
P16	*GATA3*	chr10:8100391	T	C	SNV	missense	p.Ile122Thr	rs1225263875	24.1	108	46	154	29.9	major
P16	*MAML2*	chr11:95826642	A	T	SNV	missense	p.Ser185Thr	rs112561533	16.4	73	31	104	29.8	major
P16	*HES1*	chr3:193855774	G	C	SNV	missense	p.Gly199Arg		23.0	70	25	95	26.3	major
P16	*APC*	chr5:112174464	A	G	SNV	missense	p.Asp1058Gly	rs148725540	23.4	100	30	130	23.1	major
P16	*FLCN*	chr17:17119778	T	C	SNV	missense	p.Ser406Gly	rs528541881	26.3	114	33	147	22.5	major
P16	*MSI2*	chr17:55752446	G	A	SNV	missense	p.Ala302Thr	rs145489286	24.0	155	44	199	22.1	major
P16	*PTPRB*	chr12:70925921	A	G	SNV	missense	p.Ile2133Thr	rs768311813	26.1	88	25	113	22.1	major
P16	*SOX21*	chr13:95363815	GGCGGCGGCGGCA	G	DELETION	inframe_deletion	p.Ala160_Ala163del	rs1212471987	15.9	27	6	33	18.2	minor
P16	*LRP1B*	chr2:141200150	G	A	SNV	missense	p.Thr3446Met	rs748759183	24.7	103	21	124	16.9	minor
P16	*MUC4*	chr3:195511238	C	A	SNV	missense	p.Asp2405Tyr	rs3103956	16.2	58	11	69	15.9	minor
P16	*NFKBIE*	chr6:44232738	TGTAA	T	DELETION	frameshift	p.Thr253fs	rs755160004	33.0	103	11	114	9.7	minor
P17	*ETNK1*	chr12:22778402	A	G	SNV	missense	p.Glu102Gly	rs773294981	22.9	180	174	354	49.2	major
P17	*CREBBP*	chr16:3778282	G	A	SNV	missense	p.Leu2256Phe	rs752887380	16.4	174	182	356	48.9	major
P17	*ABL1*	chr9:133738171	C	T	SNV	missense	p.Arg210Cys	rs368646202	32.0	51	39	90	43.3	major
P17	*PAX5*	chr9:36966688	G	A	SNV	missense	p.Ser213Leu	rs137870876	23.8	92	66	158	41.8	major
P17	*TSC2*	chr16:2120559	G	A	SNV	missense	p.Ala607Thr	rs45517203	24.1	92	55	147	37.4	major
P17	*FANCC*	chr9:97912337	C	T	SNV	missense	p.Arg185Gln	rs370346767	15.8	93	50	143	35.0	major
P17	*MAPKAPK2*	chr1:206858639	A	C	SNV	missense	p.Gln22Pro		16.6	53	19	72	26.4	major
P17	*RUNX1*	chr21:36164610	T	G	SNV	missense	p.Glu395Ala		24.4	58	8	56	14.3	minor

P17	*COL14A1*	chr8:121357708	G	GCCTC	INSER-TION	frameshift	p.Gly1663fs		104	15	119	12.6	minor
P17	*COL14A1*	chr8:121357702	TGGGA	T	DELE-TION	frameshift	p.Gly1660fs		108	15	123	12.2	minor
P17	*RUNX1*	chr21:36164605	A	G	SNV	missense	p.Ser397Pro	24.4	59	7	66	10.6	minor

Abbreviations are explained in Supplementary Table S3.

Table S5. Number of somatic variants and number of major variants in and gene ontology of recurrently mutated (>1 case) genes among 17 cases with primary Sjogren's syndrome related extranodal marginal zone lymphoma of mucosa-associated lymphoid tissue.

Gene	Frequency	Number of Variants	Number of Major Variants	Gene Ontology
TTC28	4	4	0	May be involved in the condensation of spindle midzone microtubules, leading to the formation of midbody during mitosis
HES1	3	3	2	Transcriptional repressor of genes that require a bHLH protein for their transcription.
HS6ST3	3	4	1	Catalyze enzyme of heparan sulfate
MAMDC4#	3	3	1	Probably involved in the sorting and selective transport of receptors and ligands across polarized epithelia
*PAX5**	2	2	2	Transcription factor that plays a role in commitment of lymphoid progenitors to the B-lymphocyte lineage
MAPKAPK2	2	2	2	Kinase involved in cytokine production, endocytosis, reorganization of the cytoskeleton, cell migration, cell cycle control, chromatin remodeling, DNA damage response and transcriptional regulation
*TBL1XR1**	2	2	2	Involved in the recruitment of the ubiquitin/19S proteasome complex to nuclear receptor-regulated transcription units
*ID3**	2	8	5	Transcriptional regulator of basic helix-loop-helix (bHLH) transcription factors: Inhibits binding of E2A-containing protein complexes to muscle creatine kinase E-box enhancer
COL14A1#	2	3	1	Adhesive role by integrating collagen bundles into the extracellular space
CAMSAP3#	2	2	2	Key microtubule-organizing protein for biogenesis and the maintenance of zonula adherens
TMEM2#	2	2	2	Cell surface hyaluronidase that cleaves extracellular high molecular weight hyaluronan into intermediate size fragments before internalization and degradation in the lysosome
*IGLL5**	2	4	1	IG lambda like peptide not requiring somatic rearrangement for expression
SLC9B1	2	3	0	Na/H exchanger involved in pH regulation of spermatozoa
AK2	2	2	1	Catalyzes transfer of the terminal phosphate group between ATP and AMP
*APC**	2	2	0	Antagonizes the Wnt signaling pathway
MUC4#	2	2	0	Glycolysated protein of mucus covering the epithelial surface

*genes associated with lymphomagenesis, # genes involved in the extracellular surface and/or extracellular matri.x
